# Photomultiplication‐Type Organic Photodetectors for Near‐Infrared Sensing with High and Bias‐Independent Specific Detectivity

**DOI:** 10.1002/advs.202105113

**Published:** 2022-01-07

**Authors:** Shen Xing, Jonas Kublitski, Christian Hänisch, Louis Conrad Winkler, Tian‐yi Li, Hans Kleemann, Johannes Benduhn, Karl Leo

**Affiliations:** ^1^ Dresden Integrated Center for Applied Physics and Photonic Materials (IAPP) and Institute for Applied Physics Technische Universität Dresden 01187 Dresden Germany; ^2^ Department of Physical Chemistry University of Science and Technology Beijing Beijing 100083 China

**Keywords:** bias‐independent, high detectivity, near‐infrared sensing, organic photodetectors, photomultiplication

## Abstract

Highly responsive organic photodetectors allow a plethora of applications in fields like imaging, health, security monitoring, etc. Photomultiplication‐type organic photodetectors (PM‐OPDs) are a desirable option due to their internal amplification mechanism. However, for such devices, significant gain and low dark currents are often mutually excluded since large operation voltages often induce high shot noise. Here, a fully vacuum‐processed PM‐OPD is demonstrated using trap‐assisted electron injection in BDP‐OMe:C_60_ material system. By applying only −1 V, compared with the self‐powered working condition, the responsivity is increased by one order of magnitude, resulting in an outstanding specific detectivity of ≈10^13^ Jones. Remarkably, the superior detectivity in the near‐infrared region is stable and almost voltage‐independent up to −10 V. Compared with two photovoltaic‐type photodetectors, these PM‐OPDs exhibit the great potential to be easily integrated with state‐of‐the‐art readout electronics in terms of their high responsivity, fast response speed, and bias‐independent specific detectivity. The employed vacuum fabrication process and the easy‐to‐adapt PM‐OPD concept enable seamless upscaling of production, paving the way to a commercially relevant photodetector technology.

## Graphical abstract

A fully vacuum‐processed photomultiplication‐type organic photodetector for near‐infrared (NIR) sensing with high and bias‐independent specific detectivity is achieved by efficient trap‐assisted electron injection. More than 100% external quantum efficiency is exhibited at only −1 V. For the NIR light, an impressive specific detectivity ≈10^13^ Jones can almost be voltage‐independent up to −10 V, indicating their potential in state‐of‐the‐art readout electronics.

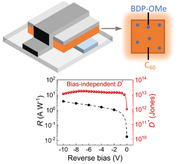

## 1 Introduction

Organic photodetectors (OPDs) are a promising optical detection technology due to the adjustable optical, electronic and mechanic properties of organic semiconductors.^[^ ^1^, ^2^, ^3^ ^]^ Thanks to advances in material science and device engineering, extensive investigations, especially in terms of dark current, enabled the rapid development of OPDs, nowadays offering comparable performance to silicon (Si) photodiodes in many parameters.^[^ ^4^, ^5^, ^6^ ^]^ Photovoltaic‐type OPDs (PV‐OPDs) typically possess moderate photoresponse, and a zero bias working condition is often needed for optimal performance, demanding more advanced and expensive readout circuits.^[^ ^7^ ^]^ Therefore, enhanced responsivity (*R*) is required, which can simplify the detection system and reduce overall costs. In this regard, photomultiplication‐type OPDs (PM‐OPDs) are anticipated for internally amplifying weak photocurrents without additional circuit components.^[^ ^8^ ^]^ In contrast to inorganic photomultiplication diodes often operated in the breakdown regime of the junction (avalanche), the operation of PM‐OPDs is mainly based on the charge tunneling injection induced by intentionally inserted traps or energetic barriers via interfacial layers.^[^ ^9^, ^10^, ^11^, ^12^ ^]^ Both strategies aim to accumulate one type of photogenerated charge carrier near the respective electrode. The electrical field caused by the trapped charge carriers induces interfacial bending of energy levels, assisting the opposite charge carrier type to be injected via tunneling. Supposing that the transit time of injected charge carriers is shorter than the lifetime of the trapped ones, a photomultiplication (PM) phenomenon occurs under illumination with an internal quantum efficiency >100%, leading to an external quantum efficiency (EQE) >100% if enough photons are absorbed.^[^ ^13^ ^]^

For solution‐processed PM‐OPDs, there have been many efforts to improve device performance and spectral window tunability. However, fewer works were reported for devices completely processed by thermal evaporation in vacuum.^[^ ^11^, ^13^, ^14^, ^15^ ^]^ Nowadays, the well‐established vacuum deposition technology is the favored fabrication technique for commercial optoelectronic devices given by superior device lifetime, precise control of layer thickness, and excellent uniformity of layer deposition.^[^ ^16^ ^]^ Furthermore, it offers great adjustability for delicate mixing ratios, vertical gradient variations, and sequential stacking of multiple layers.^[^ ^17^, ^18^ ^]^ These characteristics give vacuum evaporation distinct advantages over solution processing for the fabrication of PM‐OPDs.

While PM‐OPDs certainly will find a plethora of applications, critical performance issues need to be addressed to guarantee their successful commercialization. For example, one concern lies in the decrease of specific detectivity (*D* ^*^) under increasing bias. Typically, relatively large reverse biases are applied to operate such devices to achieve high *R*.^[^ ^19^, ^20^, ^21^, ^22^ ^]^ Meanwhile, as a result of the field‐dependent dark current,^[^ ^23^ ^]^ the PM‐OPDs suffer from high shot noise under such operating conditions. If the photogain acquired by biasing the device is accompanied by a seriously increased dark current, *D* ^*^ is likely to be sacrificed. Achieving high *R* is the main goal for PM‐OPDs, but that should not be done at the expense of *D* ^*^. Hence, finding the balance between these two counteracting characteristics is challenging but vital to realize stable *D* ^*^ under reverse bias, which is essential for the application of PM‐OPDs.

Here, we report a fully vacuum‐processed PM‐OPD with a spectral response spanning from the ultraviolet (UV) to the near‐infrared (NIR) region (300–900 nm). Based on a low donor content (4.0 wt%) in the BDP‐OMe:C_60_ material system, the device shows an EQE beyond 100% at a rather low bias of −1 V, leading to a *D* ^*^ above 10^13^ Jones. Impressively, in the NIR region, the outstanding *D* ^*^ can be well preserved as the reverse bias increases up to −10 V due to the balance between dark current and responsivity. This behavior is rarely reported for PM‐type devices. Compared to two different optimized OPDs (pin‐ and nip‐ architecture), the PM‐OPD achieves superior performance in both *R* and *D* ^*^ over a large bias range and is even comparable to Si photodiodes. In particular, these devices exhibit a synergetic performance of high *R* and fast response speed, allowing for state‐of‐the‐art readout electronics, which might speed up the adaption of these devices in commercial applications.

## 2 Results

**Figure 1:**
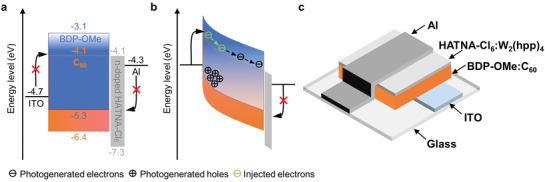
Energy diagram of PM‐OPDs investigated in this work a) in the dark at flat band condition and b) under illumination at reverse bias. c) Schematic device structure.

The BDP‐OMe:C_60_ system is investigated, given its broad absorption from the UV to NIR region (Figure S1, Supporting Information). We choose a low BDP‐OMe content to reduce the percolation paths substantially and thereby introduce hole traps. In **Figure** **1**a–c, the schematics of the PM‐OPD under dark and light as well as a layer sequence comprising ITO/BDP‐OMe:C_60_ (4.0:96.0 wt%, 400 nm)/HATNA‐Cl_6_:W_2_(hpp)_4_/Al are shown. HATNA‐Cl_6_:W_2_(hpp)_4_ is used as the electron transport layer and hole blocking layer between the active layer and the Al electrode to facilitate electron transport and reduce the reverse dark current.^[^ ^24^ ^]^ In the absence of illumination, the dark current is small because of the large charge injection barrier (>0.6 eV) under reverse bias. Under illumination, a strong hole trapping effect occurs due to the absence of a percolation network for photogenerated holes. The trapped holes shift the highest occupied molecular orbital (HOMO) of C_60_ upward close to the anode. At a small reverse bias, the electron injection barrier on the anode side becomes thin enough such that electrons can tunnel into the active layer. Accordingly, the ITO/BDP‐OMe interface acts as an optoelectronic “valve” for electron injection, and incident photons can switch on this “valve,” ideally leading to an EQE above 100%.

**Figure 2:**
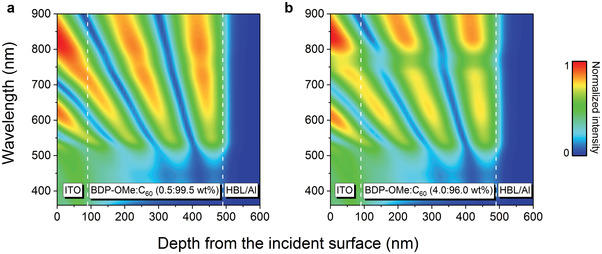
Optical field distribution of PM‐OPDs a) at 0.5 wt% donor concentration and b) at 4.0 wt% donor concentration. HBL represents the hole blocking layer of HATNA‐Cl_6_:W_2_(hpp)_4_ (3 wt%, 10 nm).

From previously reported works, the mixing ratio of the photoactive layer plays a key role for well‐performing PM‐OPDs.^[^ ^25^, ^26^, ^27^ ^]^ It has been noticed that a low concentration of one material type (donor or acceptor) is necessary to trigger PM in polymeric systems, while fewer studies are available for small molecule based devices. Zhang et al. demonstrated that hole transport is no longer limited at around 5 wt% donor content for the TAPC:C_60_ material system.^[^ ^28^ ^]^ Therefore, we employ a 4.0 wt% donor concentration to fabricate our devices. Aiming to explore the mechanism under extreme circumstances, devices comprising 0.5 wt% donor concentration are also investigated. The optical field distribution with the above‐described layer configuration is first simulated via the transfer matrix method (TMM). The results, shown in **Figure** **2**, reveal short incident wavelengths (<550 nm) are strongly absorbed in the device in both cases. Meanwhile, the 4.0 wt% device extends the additional absorption to the NIR region. This observation can be attributed to the increased donor content since BDP‐OMe is an excellent NIR absorber, shown in Figure S2 (Supporting Information). In Figure S3 (Supporting Information), the optical field distributions at two different wavelengths (460 and 770 nm) are provided for straightforward comparison.

**Figure 3:**
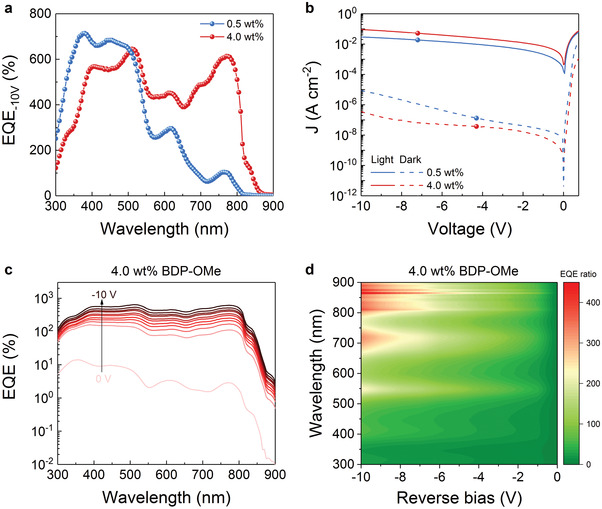
a) EQE spectra of PM‐OPDs based on BDP‐OMe:C_60_ material system at different donor concentrations under −10 V. b) *J–V* characteristics of PM‐OPDs based on BDP‐OMe:C_60_ material system at different donor concentrations. The light current was measured under 100 mW cm^–2^ illumination. c) Voltage‐dependent EQE spectra of the device comprising 4.0 wt% BDP‐OMe with a voltage step of −1 V. d) The EQE ratio of PM‐OPD at a different bias to zero bias.

To further investigate the concentration dependence, the EQE at a given reverse bias (−10 V) was measured, and the results are depicted in **Figure** **3**. At −10 V, the EQE exceeds 100% for 0.5 wt% devices, indicating that a sufficient hole accumulation can be formed at the anode at such a low doping concentration in this donor‐acceptor system, leading to increased electron injection. Benefiting from the absorption of the donor, the 4.0 wt% device shows an amplification over 600% at 780 nm without a noticeable decrease in the short wavelength range. As addressed in many other works, the dark current characteristics rather than the photoresponse performance establish the limit for detecting faint light.^[^ ^29^, ^30^ ^]^ For PM‐OPDs, low dark currents are challenging to achieve since a relatively large driving voltage is usually applied to achieve high *R*, often leading to increased noise currents simultaneously. To reduce the dark current, the good selectivity of the n‐doped contact (HATNA‐Cl_6_:W_2_(hpp)_4_) is used based on its low HOMO. As discussed by Zheng et al., highly doped transport layers lead to a lateral leakage current flow, which effectively increases the active area of organic diodes, thereby giving rise to a higher dark current density.^[^ ^31^ ^]^ In order to avoid these unwanted leakage currents, the deposition of the doped layers is conducted with the aid of structured shadow masks as suggested in the same work.^[^ ^31^ ^]^ Additionally, the doping concentration is simultaneously optimized such that selective ohmic contacts and reduced lateral current flow are concomitantly achieved. As shown in Figure 3b, the dark current density (*J*_d_) is notably reduced by increasing the donor concentration and reaches as low as 3.6 × 10^–7^ A cm^–2^ at −10 V, implying a very low shot noise. Meanwhile, the photocurrent increases more than one order of magnitude along with increased reverse bias in both cases. Particularly, a high on/off ratio, approaching 10^6^ at 100 mW cm^–2^, can be well preserved for the 4.0 wt% device (Figure S4, Supporting Information). Since 4 wt% of donor molecules gives us reliable performance with a low dark current and a high EQE in the NIR spectral region, we focus on this concentration in the upcoming discussion.

The voltage‐dependent EQE is depicted in Figure 3c. At 0 V, a maximum EQE of 2.9% is achieved in the NIR region. This low value can be explained by the interrupted percolation path for holes and a reduced charge separation probability at this concentration. Clearly, no PM is observed. After increasing the reverse bias to −1 V, the EQE rises beyond 100% rapidly, suggesting the existence of an internal amplification mechanism at such a slight reverse bias. As we further increase the reverse bias to −10 V, the EQE goes above 600% in the NIR region, which is ascribed to the synergetic effect of increased donor absorption and pronounced PM phenomenon, with the highest *R* approaching 4 A W^–1^. The voltage‐dependent *R* is shown in Figure S5 (Supporting Information). To quantify the voltage influence on the PM phenomenon, the ratio of EQE at a different bias to that at zero bias is shown in Figure 3d. Though a higher negative bias can be applied for a further increased EQE, considering *D* ^*^, the favored operation regime of this PM‐OPD is within −10 V, which will be discussed in the following section.

**Figure 4:**
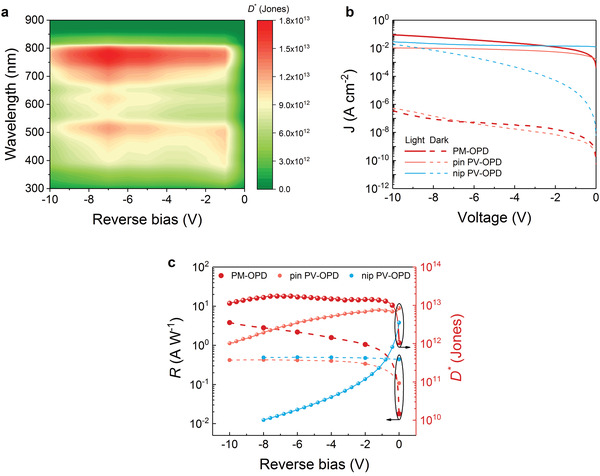
a) Shot‐noise‐limited *D* ^*^ under reverse biases of the device comprising 4.0 wt% BDP‐OMe. b) *J–V* characteristics in the dark and under 100 mW cm^–2^ illumination of two different optimized PV‐OPDs (pin‐ and nip‐architecture) and optimized PM‐OPD. c) Comparison of the same devices shown in (b) in terms of *R* and *D* ^*^. The *R* is calculated from the EQE measured at different voltages and fitted with a polynomial function at 780 nm, from which the *D* ^*^ is computed.

The specific detectivity *D* ^*^, depicting the sensitivity of a photodetector to weak optical signals,^[^ ^32^ ^]^ is given by

(1) $$D^{*}=\frac{q\lambda \mathrm{EQE}}{\mathrm{hc}S_{n}}\;\left(\text{cm}\;{\mathrm{Hz}}^{1/2}\;W^{-1}\;\text{or}\;\text{Jones}\right)$$

where *q* is the elementary charge*, λ* is the wavelength, *h* is the Planck constant, and *c* is the speed of light. In the absence of frequency‐dependent noise components, the noise spectral density (*S*_n_) reads^[^ ^33^ ^]^

(2) $$S_{n}=\sqrt{\left(2qJ_{d}+\frac{4k_{B}T}{R_{\mathrm{sh}}}\right)}\;\left(A\;cm^{-1}\;Hz^{-1/2}\right)$$

in which *J*_d_ is the dark current density, *k*_B_ is the Boltzmann constant, *T* is the temperature, and *R*_sh_ is the shunt resistance normalized by the area (Ω cm^2^) extracted from the inverse of the derivative of *J*_d_–*V* curve close to 0 V. In general, OPDs are operated in two modes: self‐powered mode (0 V) and extraction mode (under reverse bias).^[^ ^34^, ^35^ ^]^ In the case of extraction mode, which is the operating mode of PM‐OPDs, shot noise is considered as the dominant contributor to the *S*_n_ and constrains *D* ^*^ of the device.^[^ ^19^, ^36^, ^37^ ^]^ The *D* ^*^ spectra calculated from *S*_n_ under reverse biases are presented in **Figure** **4**a. The synergetic effect arising from low dark currents and the PM‐enhanced EQE enables outstanding *D* ^*^ over 10^13^ Jones. Remarkably, the high *D* ^*^ is rather stable within the scope of −10 V for NIR light. More detailed information is shown in Figure S6 (Supporting Information). We confirmed this trend for our PM‐OPDs by measuring the noise spectral density at different reverse biases (Figure S7, Supporting Information) and comparing the shot‐noise‐limited D^*^ to the values determined from the experimentally measured *S*_n_.^[^ ^30^, ^38^, ^39^ ^]^ The measured *S*_n_ shows good agreement to the shot component, being slightly higher than the latter due to other sources of noise. Nonetheless, the impact on *D* ^*^ is minor, as shown in Figure S8 (Supporting Information). This property is crucial, especially for PM‐OPDs, otherwise reaching an improved photoresponse at the expense of *D* ^*^ is not intended.

Most of the reported work proposes different solutions for EQE enhancement, sometimes neglecting the major role played by the dark current. As discussed before, an increased reverse dark current indicates an increased noise spectral density, which sets the limitation of the essential performance parameter *D* ^*^ and determines whether PM‐OPD can be an alternative to comparable PV‐OPDs. To have a comprehensive comparison, two different PV‐OPDs (pin‐ and nip‐architecture) are considered (Figure 4b). The pin PV‐OPD is fabricated comprising the same concentration with an additional BPAPF hole transport layer (HTL). The nip device is built on the BDP‐OMe:C_60_ active blend but with a standard, optimized concentration and stack combination for an efficient PV‐OPD with high EQE. The detailed device structures can be found in Table S1 (Supporting Information).

In contrast to the pin PV‐OPD, the photocurrent density of the PM‐OPD reaches 91 mA cm^–2^ at −10 V while owing a similar dark current. This finding suggests that the thin HTL (7 nm) does not affect the dark current much but forbids the PM effect. Compared with the nip PV‐OPD, the PM‐OPD offers better performance under reverse bias in light and dark conditions. Since a thin active layer is employed in the nip PV‐OPD, the enormously increased dark current induced by high reverse bias is inevitable. In order to make a fair comparison in our study, we utilize the calculated shot noise to predict the upper limit of *D* ^*^ under the reverse bias of all investigated devices. We first measured the voltage‐dependent EQE of these PV‐OPDs (Figure S9, Supporting Information) and calculated the corresponding *R* at 780 nm. Then we estimate *R* values at each voltage by utilizing a polynomial fitting function, see Figure 4c. The voltage‐dependent *D* ^*^ can be plotted according to Equation (1) and Equation (2) from the fitted *R*. The same procedure is used for the PM‐OPD. As shown in Figure 4c, increasing *R* by simply biasing is possible but not enough to achieve high *D* ^*^, especially for PV‐OPDs since *R* is weakly dependent on the applied bias. However, the dark current usually rises by orders of magnitude as a function of applied bias. Given this trade‐off, the selection of the operation region is essential. For our PM‐type device, in the most advantageous operation region, where the effect of the dark current does not overwhelm the enhancement in *R*, an impressive *D* ^*^ above 10^13^ Jones can be realized, superior to *D* ^*^ provided by the pin/nip PV‐OPDs and comparable to results for the best reported PM‐OPDs in the literature.^[^ ^19^, ^40^, ^41^, ^42^ ^]^ In contrast to the two PV‐OPDs whose *D* ^*^ decreases with increased reverse bias, the outstanding *D* ^*^ of the PM‐OPD can be maintained up to −10 V. The ideal balance between dark current and *R* in PM‐type devices is rarely reported. Besides that, the employed vacuum deposition of the PM‐OPDs offers the possibility of straight upscaling of the manufacturing and commercialization since thermal evaporation is the most established production technique for organic optoelectronic devices (e.g., organic light‐emitting diodes and organic solar cells). Whether the bias‐independent *D* ^*^ is an intrinsic consequence for our specific PM system or can be achieved generally is still unclear. Further research is needed to clarify the relationship between dark current and *R* in this device class.

**Figure 5:**
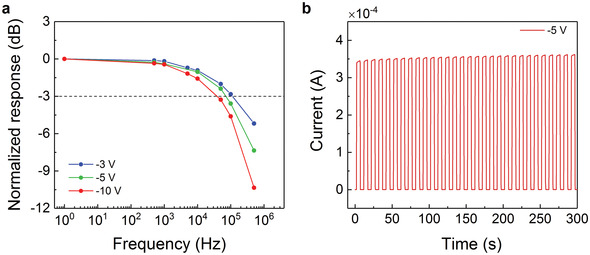
a) Normalized frequency response of PM‐OPD comprising 4.0 wt% BDP‐OMe at an illumination wavelength of 780 nm under different biases. The −3dB point is specified with the dashed line. b) Time‐dependent photoresponse of the same device operated in the presence of a white light source (0.1 Hz) under −5 V.

The frequency response is another important metric of PM‐OPDs, typically limited by the trapping or de‐trapping dynamics in these devices.^[^ ^43^, ^44^ ^]^ **Figure** **5**a shows the voltage‐dependent −3 dB cutoff frequency (*f*_−3dB_) of the PM‐OPDs, which is reduced from 112 to 39 kHz along with the increased reverse bias from −3 to −10 V. The trend is consistent with the correlation of slower response speed or narrower −3 dB bandwidth along with increased gain.^[^ ^7^ ^]^ However, the achieved values are still comparable to well‐performing PM‐OPDs reported so far and are sufficient for video‐frame rate imaging applications and compatible with state‐of‐the‐art readout electronics.^[^ ^32^ ^]^ Moreover, the time‐dependent photoresponse of the PM‐OPD measured in the presence of a white light source is shown in Figure 5b. In this measurement, the PM‐OPD was biased at −5 V for more than 5 minutes. The ON and OFF states remain highly stable during the rapid switch from illumination to dark, confirming good stability and reversibility of our PM‐OPDs. The linear dynamic range (LDR) of the PM‐OPD is demonstrated in Figure S10 (Supporting Information). Under −5 V, four orders of magnitude linearity in the photoresponse is observed.

## 3 Conclusion

In this work, a fully vacuum‐processed PM‐OPD, comprising 4.0 wt% BDP‐OMe donor concentration, is reported. Due to the efficient accumulation of holes caused by the reduction of percolation paths, a significant increase in EQE is observed at a very small reverse bias, resulting in an outstanding *D* ^*^ to values higher than 10^13^ Jones. To get a pronounced photoresponse, a large reverse bias is usually applied to PM‐OPDs. A high *D* ^*^ is difficult to preserve since the dark current is field‐dependent, generally setting the performance limitation. In the reported BDP‐OMe:C_60_ material system, the superior *D* ^*^ is almost voltage‐independent and impressively stable within −10 V (NIR spectral range). Furthermore, two kinds of corresponding photodetectors (pin‐ and nip‐ architecture) are considered for comparison. The PM‐OPD outperforms in terms of *R* and *D* ^*^ in a large bias range, demonstrating the advantage of this device class. Endowed with a fast response speed, high *R*, and Si‐level *D* ^*^, the PM‐OPDs imply a great potential in many applications, such as single‐photon detection, good imaging function without any current preamplifier, and easy integration with state‐of‐the‐art readout electronics. The vacuum fabrication method also gives PM‐OPDs a chance for facile commercial feasibility.

## 4 Experimental Section

### Device Fabrication

The devices were fabricated according to the previous work of the authors of this paper. The description is reproduced here for completeness.^[^ ^45^ ^]^ All OPDs investigated in this work are constructed by a thermal evaporation vacuum system with a base pressure of less than 10^−7^ mbar. Before deposition, ITO substrates (Thin Film Devices Inc., USA) are cleaned for 15 min in different ultrasonic baths with NMP solvent, deionized water, and ethanol, followed by O_2_ plasma for 10 min. The organic materials are purified 1 or 2 times via thermal sublimation. The device stacks of OPDs and the full name of the materials are documented in Table S1 (Supporting Information). A series of shadow masks and mobile shutters are utilized to control device layout and thickness variation. The 6.44 mm^2^ effective active area is defined by the geometrical overlap of the bottom and top contact. After fabrication, all devices are encapsulated by gluing a transparent glass on top of the device utilizing an epoxy resin (Nagase ChemteX Corp., Japan) cured by UV light. To hinder degradation, a moisture getter (Dynic Ltd., UK) is inserted between the top contact and the glass.

### Device Characterization

The *EQE spectra* under an operation voltage from 0 to −10 V are measured with a lock‐in amplifier (Signal Recovery SR 7265, USA) under monochromatic illumination (Oriel Xe Arc‐Lamp Apex Illuminator combined with Newport Cornerstone 260 1/4 m monochromator, USA) using a calibrated monocrystalline Si reference diode (Hamamatsu S1337, Japan, calibrated by Fraunhofer ISE). An aperture is used to minimize edge effects and define an exact photoactive area (2.997 mm^2^). *Illuminated J–V characteristics* are recorded with a source‐measuring unit (SMU) (Keithley Instruments Keithley 2400, USA) under ambient conditions. The devices were illuminated at an intensity of 100 mW cm^−2^ provided by a sun simulator (Solarlight Company, USA). The intensity is calibrated by a Hamamatsu S1337 Si photodiode. *Dark J–V characteristics* are measured with a high‐resolution SMU (Keithley Instruments Keithley 2635, USA). The *noise spectral densities* of the devices under different reverse biases are calculated by applying Welch's method on the time‐dependent dark currents, which were measured using an oscilloscope (Tektronix DPO7354C, USA) connected to a low noise current‐voltage amplifier (Femto DLPCA‐200, Germany). The best efforts were devoted to optimize the measurement setup by using the aforementioned low‐noise amplifier, its internal bias supply, shortening the connection cable, as well as shielding the measurement box. For obtaining the −*3dB bandwidth* of the PM‐OPD, one light emitting diode (LED) with an emission peak wavelength at 780 nm (Roithner Lasertechnik GmbH, Germany) is used as a light source with an oscilloscope (Tektronix DPO7354C, USA) connected with a low noise current‐voltage amplifier (Femto DLPCA‐200, Germany) to determine the response signal. A power supply is connected to the device to provide corresponding voltages at the same time. The *time‐dependent photoresponse* is measured under a white LED (Luxeon K2 with TFFC, the Netherlands) in connection with a signal generator to control the modulation of the light source and the above‐mentioned high‐resolution SMU (Keithley Instruments Keithley 2635, USA) to determine the time‐dependent current signal of the device. The *LDR* is obtained using a LED light source with an illumination wavelength peak at 600 nm (Roithner Lasertechnik GmbH, Germany). The photocurrent is recorded with a high‐resolution SMU (Keithley Instruments Keithley 2635, USA) and calibrated with a Si photodiode.

*Optical simulations* are performed using an in‐house developed optical simulation tool based on the TMM. The optical constants of all layers are obtained by variable angle spectroscopic ellipsometry utilizing an EP4 imaging ellipsometer (Accurion GmbH, Germany). An optical model is applied in order to extract the complex refractive indices.

## Conflict of Interest

The authors declare no conflict of interest.

## Supporting information

Supporting Information

## Data Availability

The data that support the findings of this study are available from the corresponding author upon reasonable request.
