# Supplementary material for: Photomultiplication‐Type Organic Photodetectors for Near‐Infrared Sensing with High and Bias‐Independent Specific Detectivity
Source: Adv Sci (Weinh). 2022 Jan 7;9(7):2105113. doi: 10.1002/advs.202105113 (PMC8895121; doi:10.1002/advs.202105113)
Supplement: Supplementary file 1 — Supporting Information [file ADVS-9-2105113-s001.pdf]

WILEY-VCH

ADVANCED SCIENCE
Open Access

Supporting Information

for *Adv. Sci.*, DOI: 10.1002/advs.202105113

Photomultiplication-Type Organic Photodetectors for Near-Infrared Sensing with High and Bias-Independent Specific Detectivity

Shen Xing[*], *Jonas Kublitski, Christian Hänisch, Louis Conrad Winkler, Tian-yi Li, Hans Kleemann, Johannes Benduhn*[*] *and Karl Leo*[*]

Supporting Information

Photomultiplication-Type Organic Photodetectors for Near-Infrared Sensing with High and Bias-Independent Specific Detectivity

Shen Xing, Jonas Kublitski, Christian Hänisch, Louis Conrad Winkler, Tian-yi Li, Hans Kleemann, Johannes Benduhn* and Karl Leo**

Supporting Figures

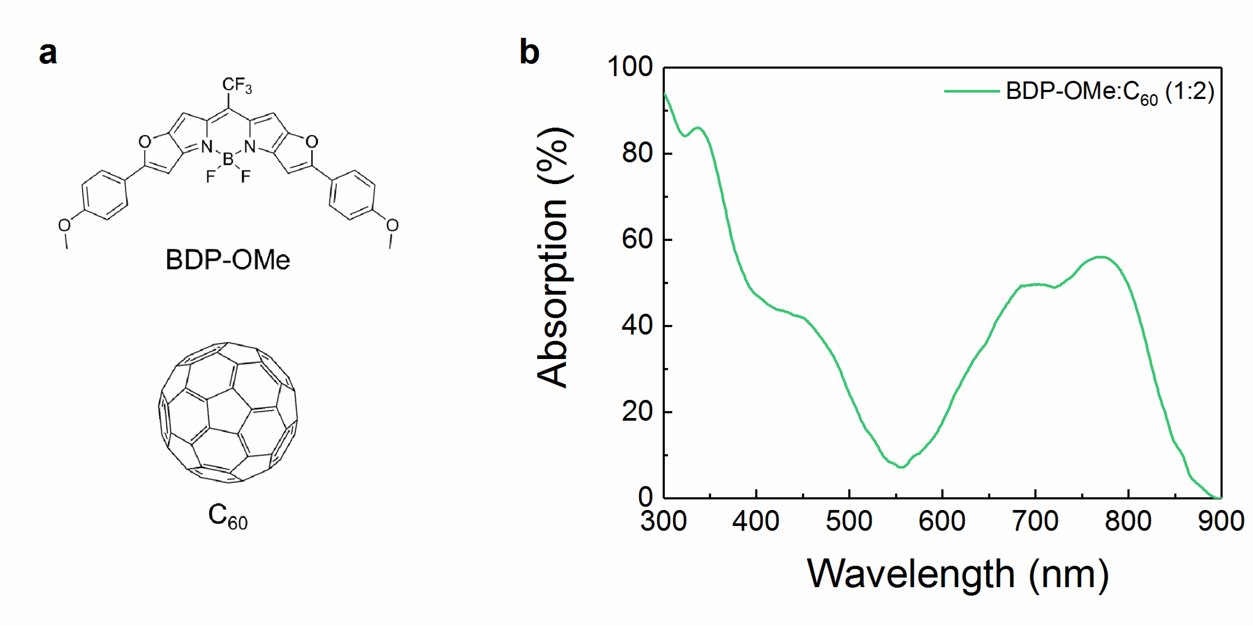

Figure S1. (a) Chemical structures of selected donor and acceptor material. (b) Simulated absorption spectrum of BDP-OMe:C_{60} blend based on measured optical constants. The simulated structure is glass (1 mm) / BDP-OMe:C_{60} (1:2, 100 nm).

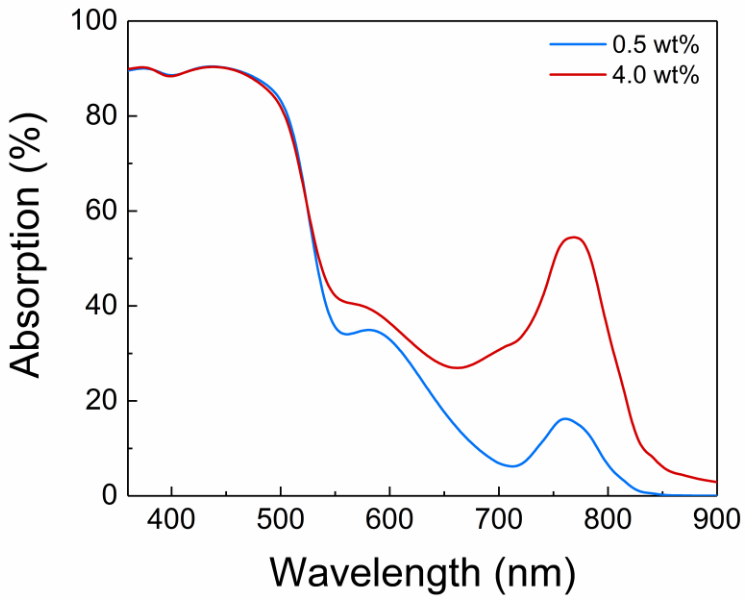

Figure S2. Simulated absorption spectra based on measured optical constants at different donor concentrations. The simulated structure is glass (1 mm) / BDP-OMe:C_{60} (400 nm) at two different concentrations, listed in the legend.

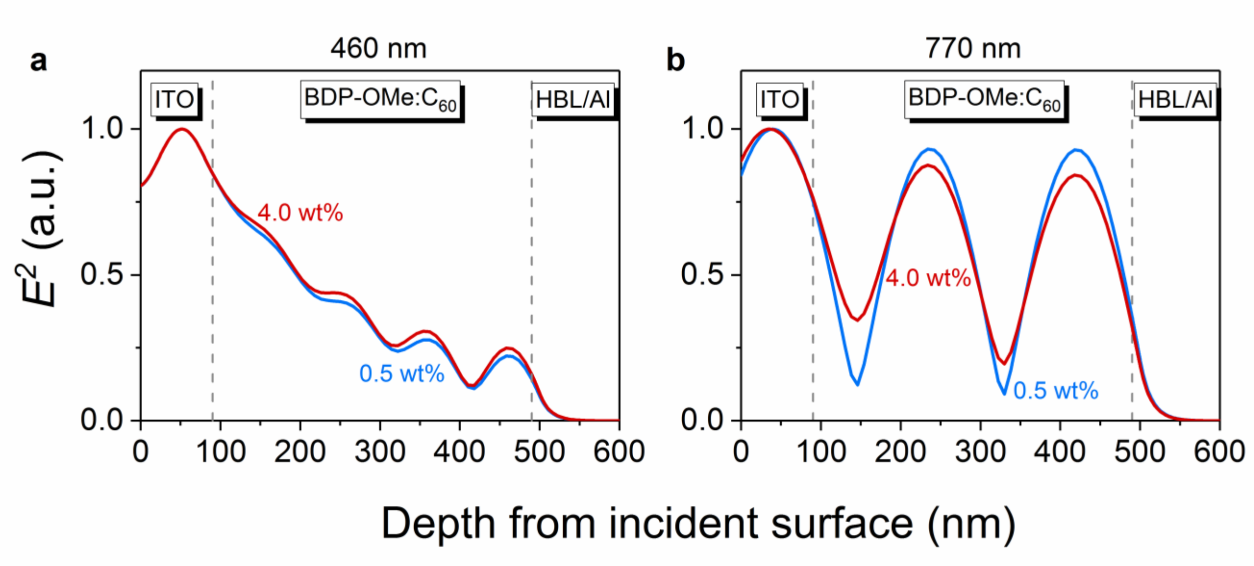

Figure S3. Optical field distribution profiles of PM-OPDs at two different wavelengths of (a) 460 nm and (b) 770 nm.

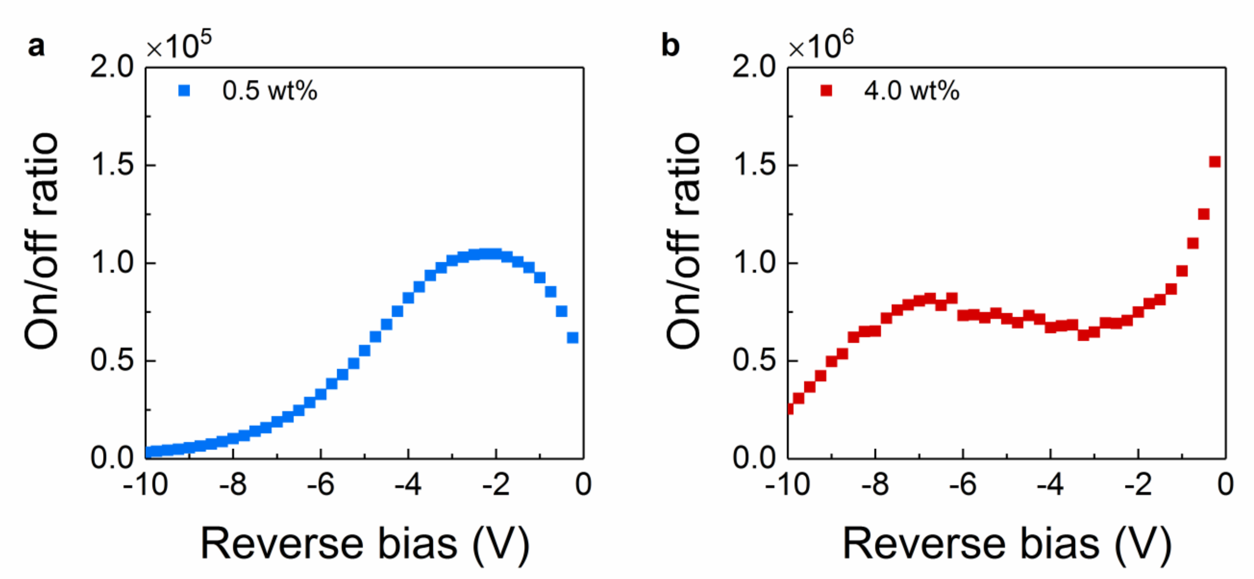

Figure S4. On/off ratio of PM-OPDs (a) at 0.5 wt% donor concentration and (b) at 4.0 wt% donor concentration. The photocurrent ("on") was measured under white light illumination at 100 mW cm^{-2}.

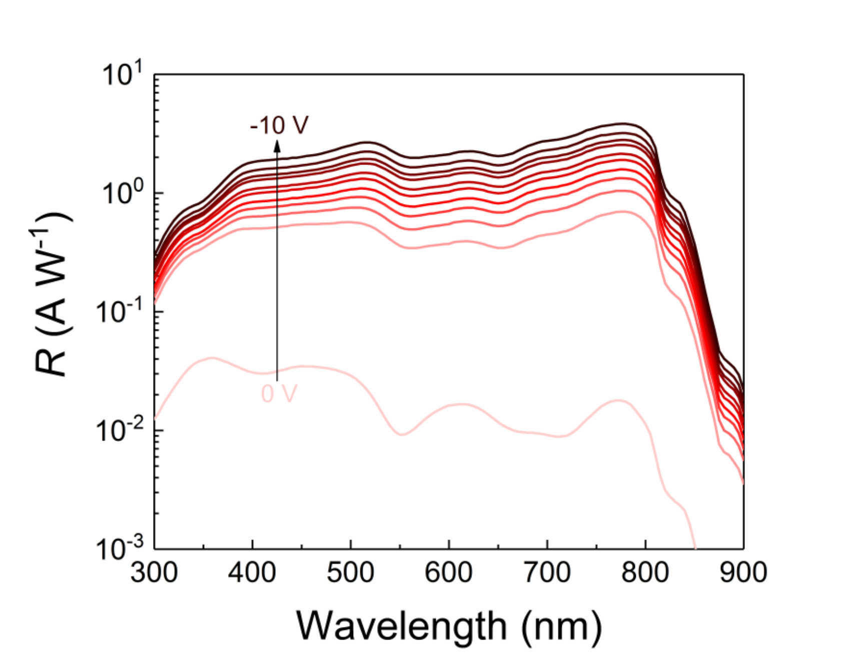

Figure S5. Voltage-dependent *R* of the device comprising 4.0 wt% BDP-OMe with a voltage step of -1 V.

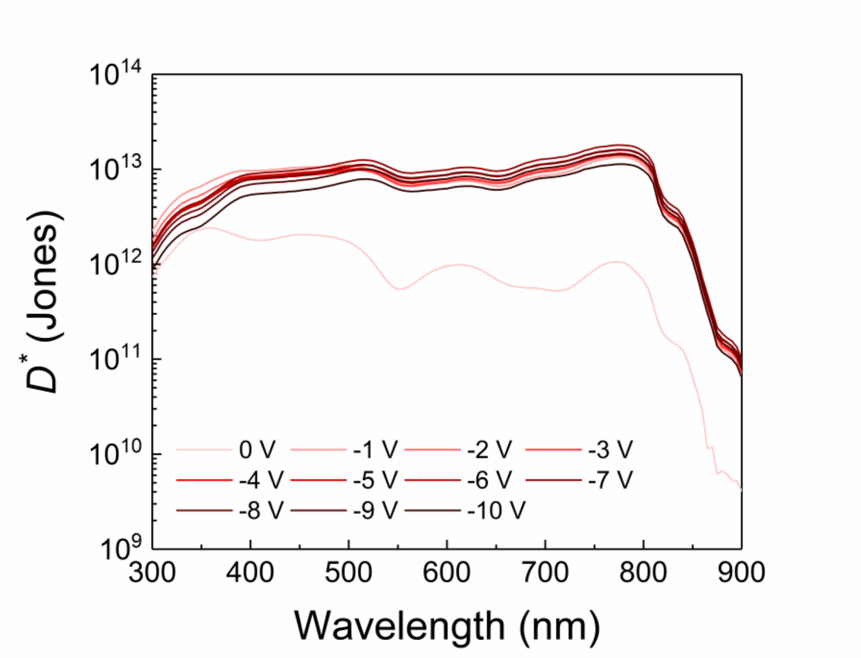

Figure S6. Voltage-dependent D^* of the device comprising 4.0 wt% BDP-OMe with a voltage step of -1 V.

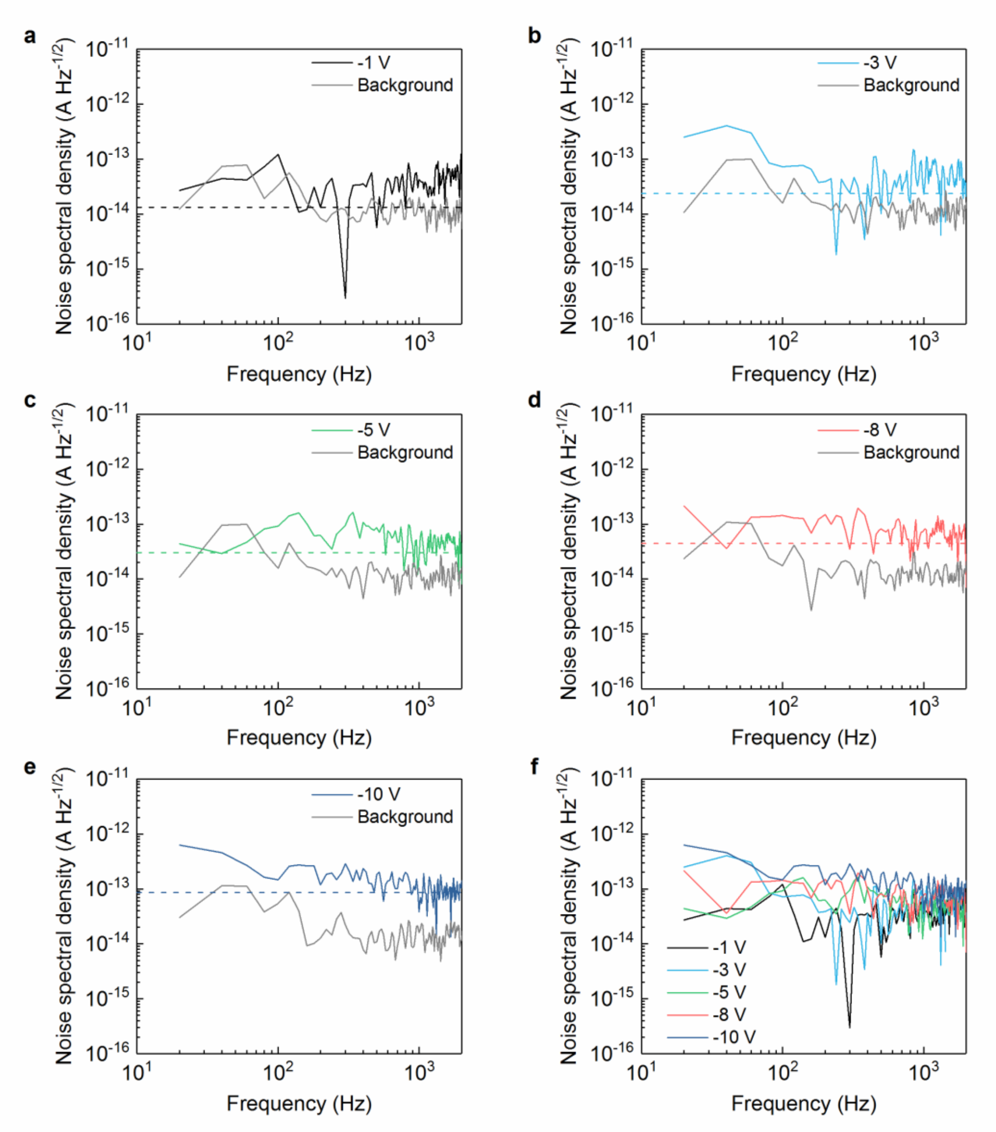

Figure S7. Measured noise spectral density of the device comprising 4.0 wt% BDP-OMe at a reverse bias of (a) -1 V, (b) -3 V, (c) -5 V, (d) -8 V, and (e) -10 V as well as the corresponding setup limitation shown as the grey solid line. The theoretical shot noises under different reverse biases are specified with the dashed lines. (f) Summary of measured bias-dependent noise spectral densities of the device comprising 4.0 wt% BDP-OMe.

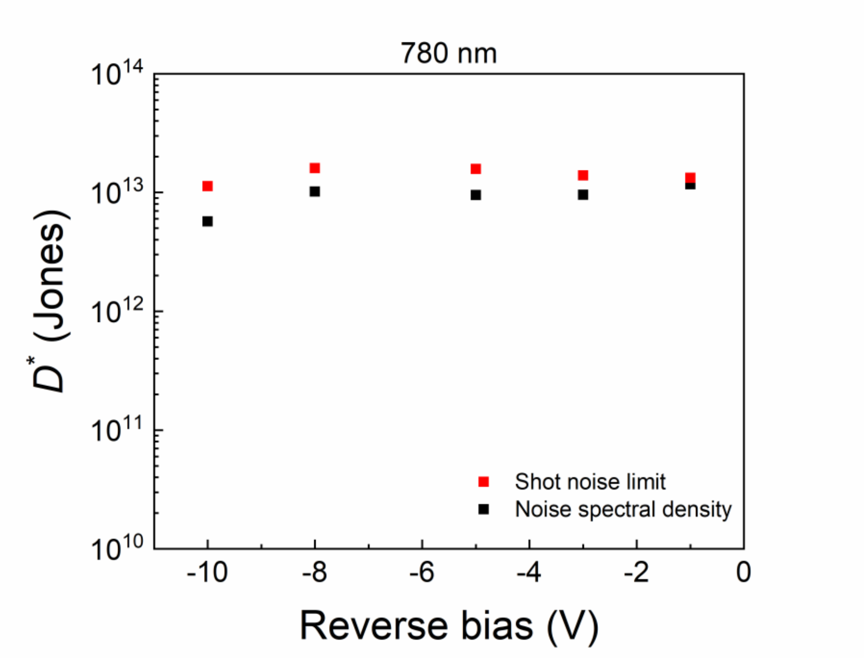

Figure S8. Calculated D^* for the photoresponse at 780 nm from the shot noise limit (red squares) and the measured noise spectral density (black squares) of the device comprising 4.0 wt% BDP-OMe under reverse bias.

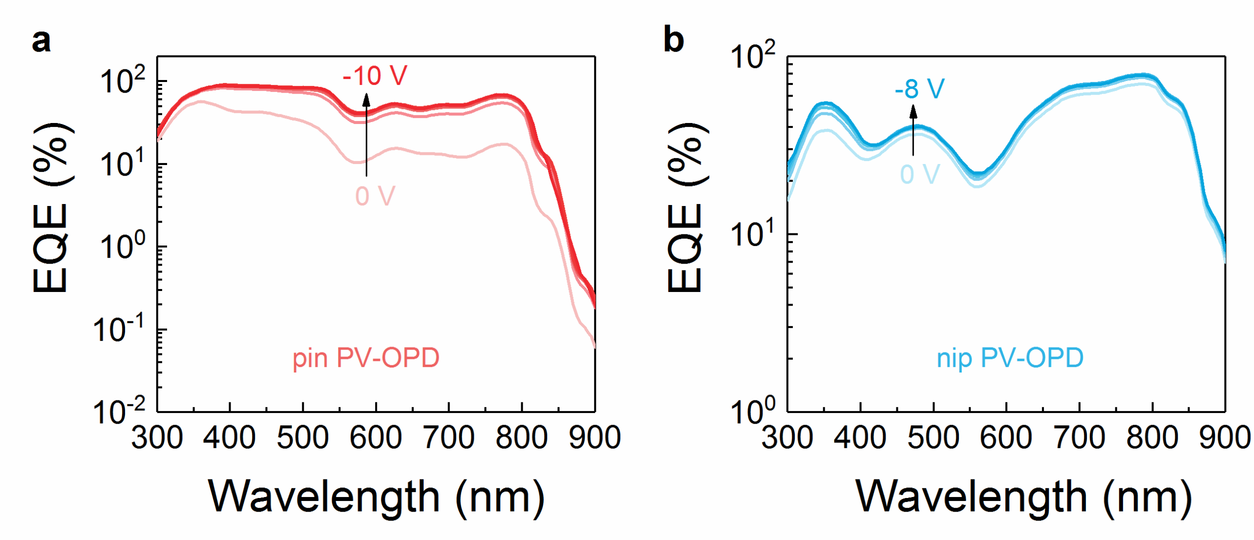

Figure S9. Voltage-dependent EQE of optimized (a) pin PV-OPD and (b) nip PV-OPD with a voltage step of -2 V.

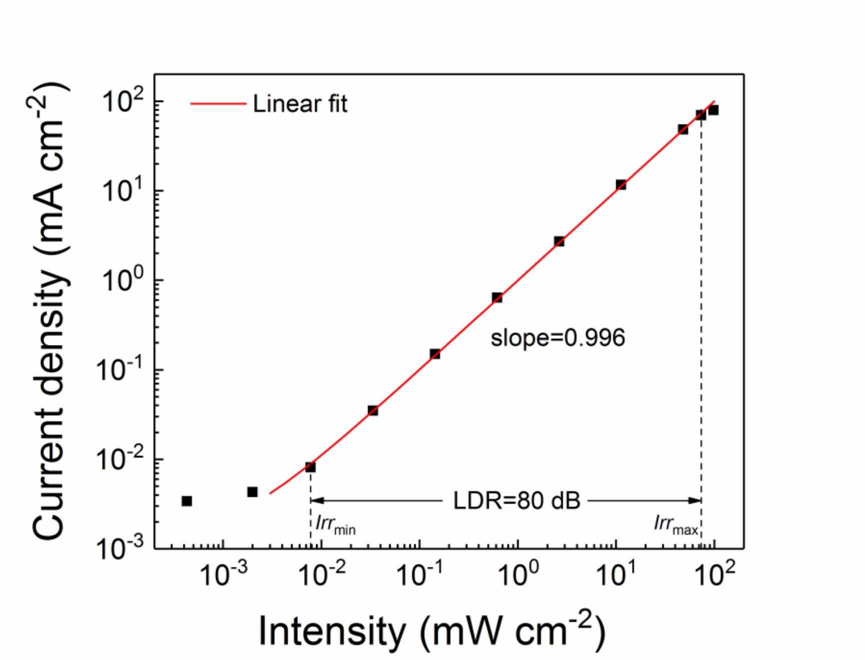

Figure S10. LDR of the device comprising 4.0 wt% BDP-OMe under 600 nm light illumination and an applied voltage of -5 V. The LDR is calculated via the equation: LDR=20 logIrr_{max}/Irr_{min} (dB), where Irr_{max} and Irr_{min} are the maximum and minimum light intensity that the photocurrent of an OPD follows a linear dependence on light intensity.

Supporting Tables

Table S1. Device stacks of all investigated OPDs. For the devices, the following materials are used: the electrode materials: ITO (Thin Film Devices Inc., USA) and Al (Kurt J. Lesker Company Ltd., USA), the donor material: BDP-OMe (CF_3-BODIPY) (TU Dresden, Germany), the acceptor material: C_{60} (buckminster fullerene) (Lumtec Corp., Taiwan), the transport layer materials: BPAPF (9,9-bis[4-(N,N-bis-biphenyl-4-yl-amino)phenyl]-9H-fluorene) (Lumtec Corp., Taiwan), HATNA-Cl_6 (2,3,8,9,14,15-hexachloro-5,6,11,12,17,18-hexaazatrinaphthylene) (Lumtec Corp., Taiwan) and Bis-HFl-NTCDI (N,N-Bis(fluoren-2-yl)-naphthalenetetracarboxylic diimide) (TU Dresden, Germany), and the dopants: NDP9 (Novaled Dopant P-Side No. 9) (Novaled GmbH, Germany), and $W_2(hpp)_4$ (Ditungsten tetra(hexahydropyrimidopyrimidine)) (Novaled GmbH, Germany).

**OPD**	**Stack**
PM-OPD	Glass (1 mm) / ITO (90 nm) / BDP-OMe:C_{60} (0.5:99.5 wt%, 400 nm) / HATNA-Cl_6:$W_2(hpp)_4$ (3 wt%, 10 nm) / Al (100 nm)
	Glass (1 mm) / ITO (90 nm) / BDP-OMe:C_{60} (4.0:96.0 wt%, 400 nm) / HATNA-Cl_6:$W_2(hpp)_4$ (3 wt%, 10 nm) / Al (100 nm)
pin PV-OPD	Glass (1 mm) / ITO (90 nm) / BPAPF (7 nm) / BDP-OMe:C_{60} (4.0 wt%:96.0 wt%, 400 nm) / HATNA-Cl_6:$W_2(hpp)_4$ (3 wt%, 10 nm) / Al (100 nm)
nip PV-OPD	Glass (1 mm) / ITO (90 nm) / Bis-HFl-NTCDI:$W_2(hpp)_4$ (7 wt%, 5 nm) / C_{60} (15 nm) / BDP-OMe:C_{60} (1:2 substrate temperature at 90 °C, 50 nm) / BPAPF (5 nm) / BPAPF:NDP9 (10 wt%, 50 nm) / NDP9 (1 nm) / Al (100 nm)
